# Safety and efficacy of targeted hyperthermia treatment utilizing gold nanorod therapy in spontaneous canine neoplasia

**DOI:** 10.1186/s12917-017-1209-y

**Published:** 2017-10-02

**Authors:** Elizabeth M. Schuh, Roberta Portela, Heather L. Gardner, Christian Schoen, Cheryl A. London

**Affiliations:** 1 College of Veterinary Medicine, The Ohio State University, Columbus OH AND Cummings School of Veterinary Medicine, Tufts University, North Grafton, MA USA; 2 Premier Veterinary Group, 3927 W. Belmont Ave, Chicago, IL 60618 USA; 3 Nanopartz Inc., 146 Barberry Place, Loveland, CO 80537 USA; 4 Veterinary Cancer Group, 2887 Edinger Ave, Tustin, CA 92780 USA; 5 Sackler School at Tufts University, Boston, MA USA

**Keywords:** Hyperthermia, Plasmonic photothermal therapy, Gold nanorod, Dog, Tumor

## Abstract

**Background:**

Hyperthermia is an established anti-cancer treatment but is limited by tolerance of adjacent normal tissues. Parenteral administration of gold nanorods (NRs) as a photosensitizer amplifies the effects of hyperthermia treatment while sparing normal tissues. This therapy is well tolerated and has demonstrated anti-tumor effects in mouse models. The purpose of this phase 1 study was to establish the safety and observe the anti-tumor impact of gold NR enhanced (plasmonic) photothermal therapy (PPTT) in client owned canine patients diagnosed with spontaneous neoplasia.

**Results:**

Seven dogs underwent gold NR administration and subsequent NIR PPTT. Side effects were mild and limited to local reactions to NIR laser. All of the dogs enrolled in the study experienced stable disease, partial remission or complete remission. The overall response rate (ORR) was 28.6% with partial or complete remission of tumors at study end.

**Conclusions:**

PPTT utilizing gold nanorod therapy can be safely administered to canine patients. Further studies are needed to determine the true efficacy in a larger population of canine cancer patients and to and identify those patients most likely to benefit from this therapy.

## Background

The emerging field of nanoparticles holds promise for a variety of applications including but not limited to imaging contrast enhancement, cancer treatment, drug and gene delivery, fluorescent labeling and tissue engineering [1]. Specifically, gold nanorods (NRs) have been identified as an optimal nanoparticle as they may be synthesized in bulk, have broadly tunable plasmon resonance and exhibit absorption coefficients 10^4^–10^6^-fold higher than conventional organic fluorochromes typically used for photodynamic therapy [2, 3]. While both spherical and rod gold nanoparticles exhibit a specific plasmon frequency according to their size and shape [4], only gold NRs can be engineered to near-infrared (NIR) plasmon resonance for biomedical in vivo uses [5, 6]. The critical NIR wavelength window between 650 and 900 nm allows for absorption and scatter in the optical light range making gold nanorods an excellent choice for high sensitivity photoacoustic contrast and thermal ablation of solid tumors [7, 8].

More recently, NRs have received attention as a potential photothermal agent for targeted hyperthermia with a wide safety range [3, 7]. The NRs have the ability to passively accumulate within tumors due to the chaotic and poorly developed framework of most tumor blood vessels. When administered by intravenous injection a proportion of the NRs are cleared by the reticuloendothelial system while the remaining NRs tend to become lodged in tumors. This enhanced permeability and retention or “EPR” effect is critical for both therapeutic and imaging techniques [9, 10]. One study shows that non-coated gold NRs clear the bloodstream almost immediately while polyethyleneglycol (PEG) polymers applied to the NR surface extends their half-life to 19 h due to immune system evasion, with the spleen showing the highest collection of NRs [11]. When gold NRs are coupled with visible and NIR-absorbing molecules, the pairing results in Surface-Enhanced, Resonant Raman Scattering (SERS) creating a unique spectral multiplexing density far superior to alternate methods, making them a highly suitable tool for targeted photothermal ablation [3].

A key safety concept while utilizing SERS NRs is ensuring specific targeting of tumors. For effective ablation to occur through hyperthermia in vivo*,* the enhanced NRs must demonstrate significant EPR effects to maximize anti-tumor efficacy while sparing normal tissues. Multiple mouse models have now demonstrated aggregation of NRs in xenogeneic tumors following intravenous administration [3, 12, 13]. In vivo, SERS-coated NRs permit remote photothermal tumor heating to ablative temperatures via ex vivo NIR diode lasers [3]. When these NRs are treated from an external laser source, varying degrees of success (25–57%) have been achieved in shrinking xenogeneic tumors dependent on the route in which the NRs are administered [12, 14]. Intratumoral NR administration followed by computationally derived quantitative photothermal modeling and NIR photothermal heating has recently been successfully used to treat tumors in mouse models of cancer without evidence of systemic toxicity with a 100% response rate that was durable over 50 days [13]**.** These data support the notion that notion that SERS NRs represent an alternative approach to traditional hyperthermia based cancer therapies. While the SERS nanorods have been studied extensively in mouse models of cancer, they have not been extensively tested in the setting of spontaneous disease. As such, the objectives of this clinical trial were to assess the adverse event profile of SERS NRs administered intravenously to dogs with solid tumors and to evaluate the anti-tumor activity of SERS NRs in dogs with solid tumors following treatment with a NIR diode laser.

## Methods

### Eligibility

This clinical trial was approved by the Clinical Research and Advising Committee at the College of Veterinary Medicine and the Institutional Animal Care and Use Committee (IACUC) at the Ohio State University. Informed consent was obtained from all owners prior to study enrollment and the clinical trial was performed in compliance with guidelines for conducting clinical trials in client owned animals at the College of Veterinary Medicine. Canine patients presenting to the Ohio State University Veterinary Medical Center Oncology service diagnosed with a solid tumor (carcinoma, sarcoma or mast cell tumor) that was at least 2 cm in size and amenable to repeat biopsy were eligible for enrollment. The patient may have failed standard therapy or had no other known effective therapeutic options. Alternately, the owners may have elected to enter the patient in lieu of standard of care. Prior to enrollment dogs underwent complete blood count, chemistry, urinalysis and thoracic radiograph testing. Patients were required to be over 1 year of age with adequate organ function (ALT and AST <4 times upper limit of institutional normal, bilirubin within normal limits and serum creatinine <2.5 mg/dL) and have an estimated life expectancy of at least 6 weeks. Exclusion criteria included evidence of metastatic disease, underlying cardiac disease/arrhythmias or any other serious systemic disorder incompatible with the study. Patients were required to be at least 3 weeks post any surgical intervention and not currently using any other investigational drug. If the patient was treated with prior radiation or chemotherapy, it also must have been completed these treatments at least 3 weeks prior to enrollment and be completely recovered from any acute toxicities.

### Investigational drug product and concomitant medication

The gold nanorods were supplied by Nanopartz Inc. (Loveland, CO), and were a formulation of gold nanorods with a methyl terminated polyethylene glycol coating suspended in deionized water to a concentration of 10 mg/ml. These NRs were engineered to a surface plasmon resonance of 800 nm for photothermal heating. Concomitant medications permissible for use in prevention or managing adverse events included metoclopramide, ondansetron, maropitant, famotidine, ranitidine, omeprazole, metronidazole, bismuth subsalicylate, loperamide, butorphanol, tramadol, fentanyl, diphenhydramine. Patients were eligible to receive either an NSAID (non-steroidal anti-inflammatory drug) or prednisone as needed for pain relief or swelling associated with treatment at the discretion of the primary investigator.

### Study design

A total of 7 dogs were enrolled in this study and each patient had a single tumor that was undergoing evaluation. Following screening, a baseline tumor biopsy was obtained with a 14 g needle core biopsy, processed in formalin and histopathologically evaluated through the Ohio State University Anatomic Pathology service. SERS NRs were administered intravenously at a dose of 10 mg/kg. Both plasma and serum absorbance was performed to assess NR levels at the following time points: pre-treatment, 15 min post administration, and 72 h post administration. Dogs underwent NIR diode laser treatment 72 h post administration to ensure maximal clearance of the NRs and thus reduce the possibility of off-target effects. The tumor was then heated to a minimum of 40 °C for 1–2 min; an infrared camera was used to track the heating process. Dogs were evaluated 7 days following treatment with a physical examination and on day 14 after treatment a physical examination, CBC, serum biochemistry profile. An assessment of adverse events was performed at each visit. At Day 14, dogs were eligible to undergo tumor removal. If this was not elected, then a second needle core biopsy was performed at this time point. Dogs that had surgical resections of their tumor were evaluated 2 weeks post-operatively; dogs that had second needle core biopsies were evaluated at 2 and 6 weeks post-biopsy for evidence of adverse events secondary to NR treatment.

### NR administration and assessment of serum and plasma concentrations

Gold nanorods were provided at a concentration of 10 mg/ml in 0.9% NaCl and stored at 4 °C. Prior to administration, the gold nanorod concentrate was diluted in 0.9% NaCl to a final concentration of 5 mg/ml. Nanorods were given as an intravenous infusion at a fluid rate of 50 ml/h or 250 mg nanorods/h. For example, a 25 kg dog would receive 250 mg of gold nanorods diluted in 50 ml of 0.9% NaCl given over 1 h. This dose (10 mg/kg) was based on clinical evaluation of NRs in normal beagle dogs in which doses up to 32 mg/kg (unpublished data, CARE Research LLC) were given with no adverse events. A portable UV/Vis (ultraviolet visible) spectrometer (Nanopartz fiber-optically modified Thor Labs CCS175 Compact UV/Vis spectrometer, Loveland CO 80537) was utilized to measure nanorods in the bloodstream and compared to a standard curve to estimate nanorod concentration. Briefly, serum and plasma were obtained from each patient through routine blood draw and centrifugation. The intensity of light passing through the sample was compared to the intensity of light before passing through the sample in order to calculate the absorbance. The UV/Vis spectrometer was calibrated against known standards, with a longitudinal absorption profile ranging from 700 to 950 nm. Nanorod concentration was assessed pretreatment, 15 min post treatment and 72 h post treatment in both plasma and serum. The analysis from the UV VIS was used to confirm nanorod concentration at the 72 h time point prior to NIR laser application to determine if the NRs had cleared the bloodstream prior to laser application.

### Laser application and thermal imaging

Following administration of the NRs, dogs returned 72 h later to ensure that they had cleared the bloodstream. Dogs were then sedated with butorphanol (0.2 mg/kg) and acepromazine (0.02 mg/kg) by intramuscular injection 30 min prior to application of the laser. Once sedated, dogs were positioned to provide maximal access to the tumor. The normal tissues surrounding the target area were covered and exposure to laser was minimized. Appropriate personal protective equipment including safety glasses were utilized by staff members applying the laser and observing laser application (Jenoptik laser, JOLD-30 FC-14 30 W 400 μm fiber output, 808 nm Jena, Germany 07743). The laser was then applied to the tumor until the tumor has reached a sustained temperature of 40C for 1–2 min. The temperature assessment was performed using a thermal imaging camera (RAZ-IR, Las Vegas NV 89113). It was anticipated that the total treatment time to achieve a temperature of 40 °C for 1–2 min would be approximately 5 min. The dogs were then allowed to recover from sedation and be returned to the owner.

### Evaluation of response

Determination of antitumor efficacy was based on objective tumor assessments made according to the RECIST (response evaluation criteria in solid tumors) guidelines consistent with the current guidelines [15]. Briefly, a complete response (CR) was defined as the complete disappearance of the target lesion and partial response (PR) was defined as a ≥ 30% decrease in the longest dimension of the target lesion taking as a reference the baseline longest dimension. Stable disease (SD) is defined as neither sufficient shrinkage to qualify for PR nor sufficient increase to qualify for PD taking as a reference the baseline longest dimension. The time interval to qualify for stable disease was the duration of the study (14 days). Progressive disease (PD) was defined as a ≥ 20% increase in the longest dimensions of the target lesion taking as a reference the baseline longest dimension or the appearance of 1 or more new lesions. Tumor assessments were obtained prior to treatment then day 7 and 14 post NIR laser treatment via caliper measurement of the longest diameter with photographic documentation.

### Assessment of adverse events

Dogs were evaluated for adverse events (AEs) at every study visit. AEs were defined and graded according to the published VCOG-CTCAE criteria [16].

## Results

### Demographics

Seven dogs were enrolled into this clinical trial. The median age was 9 years (range 6–13 years) and the median weight was 28.2 kg (9.1–42.1). There were 4 spayed females and 3 neutered males enrolled, with several breeds represented including two Beagles and one of each of the following: Labrador Retriever, Golden Retriever, Boston Terrier, Greyhound, and Mixed Breed. Most dogs had undergone no prior therapy (*n* = 5) while one had recurrence of disease following surgery and another had recurrence following surgery and radiation therapy. The majority of dogs had a soft tissue sarcoma (*n* = 5) and the remaining 2 dogs had a mast cell tumor (Table 1).

**Table 1:** Patient demographics

Patient	Breed	Age (yr)	Sex	Wt (kg)	Disease	Prior Treatment
1	Greyhound	12	MN	28.2	STS	Surgery
2	Mixed Breed	8	MN	37.8	STS	None
3	Boston Terrier	6	FS	11.7	STS	None
4	Beagle	9	MN	17.0	MCT	None
5	Beagle	13	FS	9.1	STS	None
6	Labrador Retriever	10	FS	38.3	MCT	None
7	Golden Retriever	7	FS	42.1	STS	Surgery and radiation

### Nanorod dosing, measurement and clearance

**Fig. 1:**
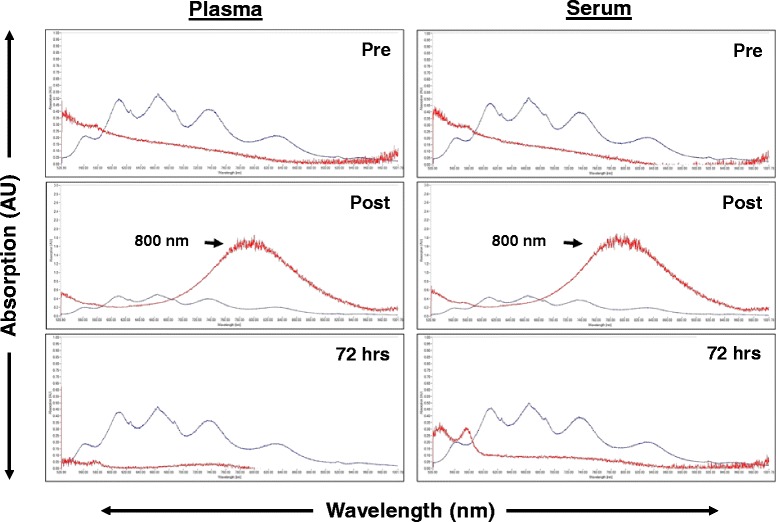
Plasma and serum NR concentrations before and after treatment. The NR absorption peaks in Patient 7 are noted by the red line; background from blood components is noted with the blue line. A reliable peak is present at 800 nm (red) 15 min post NR infusion. Clearance of the NRs from serum and plasma is seen by 72 h

Nanorod dosage was standardized at 10 mg/kg and the total dose given ranged from 91 to 410 mg reflecting variation in patient size. Nanorod absorption was measured in plasma and serum and found to be comparable between the two samples (Fig. 1). Both samples showed minimal background scatter in the pre-treatment samples, a reliable peak at 800 nm 15 min post treatment and minimal to no residual nanorod detection 72 h post administration. The nanorod concentration 15 min post treatment was variable ranging from 60 to 192 μg/ml (Table 2). Most patients showed near complete clearance of circulating nanorods by 72 h post administration (Table 2). Interestingly, one dog had evidence of persistent high plasma levels of nanorods (97 μg/ml) at 72 h post administration although NIR laser application was completed without incident.

**Table 2:** Plasma concentrations of NRs immediately and 72 h post treatment

Patient	NR Post Treatment (μg/ml)	NR 72 h (μg/ml)
1	108	12
2	120	0
3	192	97
4	160	0
5	60	6
6	72	0
7	168	0

### NIR laser application

**Fig. 2:**
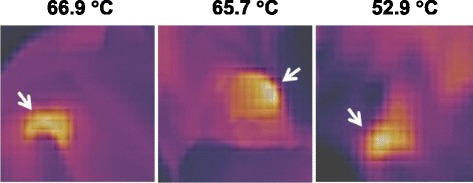
Infrared imaging of tumors during NIR laser therapy. Shown are representative images of infrared imaging performed during treatment, with the associated intratumoral temperatures as assessed by the infrared camera

After clearance of nanorods from the bloodstream was established, the NIR laser was applied to the tumors. The median treatment time was 2 min with therapy times ranging from 40 s to 4 min. All patients achieved a minimum tumor temperature of 40 °C (range 40–110 °C, Table 3) as assessed using real time monitoring with the infrared camera (Fig. 2).

**Table 3:** Thermal dose during NIR Diode laser treatment

Patient	Thermal Dose
1	75–100 ° C for 1 min
2	40–60 °C for 2 min
3	53–66 °C for 3 min
4	63–70 °C for 1 min
5	45–55 °C for 4 min
6	78–110 °C for 40 s
7	45–65 °C for 3 min

### Treatment outcome

**Fig. 3:**
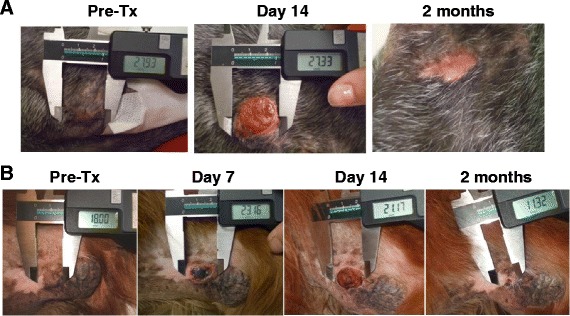
Response to NIR therapy. **a** Patient 1 experienced a durable response that lasted for approximately 8 months post treatment. **b** Patient 7 showed partial response for 77 days at which point the tumor showed mild enlargement from the previous visit and a surgical excision was elected (**b**)

All patients completed the study as expected with a varied follow up after the study period. Patients 1 and 7 experienced objective response with a CR and PR respectively showing a 28.6% objective response rate (both soft tissue sarcomas). After experiencing stable disease and having a needle core biopsy performed at the end of the study period, Patient 1 experienced a durable CR that lasted for approximately 8 months post treatment (Fig. 3a). No further surgical intervention was pursued for Patient 1. Patient 7 showed partial response for 77 days at which point the tumor showed mild enlargement from the previous visit and a surgical excision was elected (Fig. 3b). The patients that experienced stable disease (Patients 2, 3, 4, 5 and 6) had their tumors surgically removed at or near the end of the study period.

### Histopathology

Baseline and post treatment histopathology were obtained in all dogs enrolled in this study (Table 4). Tumors from Patients 1, 3, 4 and 5 showed moderate changes in response to PPTT including coagulation, edema, hemorrhage and skin necrosis evident in their post treatment biopsies. The soft tissue sarcoma from Patient 1 demonstrated necrosis involving 20% of the submitted biopsy; this dog went on to achieve a complete response to therapy. The soft tissue sarcoma from Patient 3 showed approximately 60% microscopic tumor necrosis in response to treatment but the tumor remained unchanged grossly. Patient 4’s mast cell tumor showed 40–50% necrosis with fibrin and hemorrhage in the center of the tumor and moderate to severe skin necrosis microscopically but also remained unchanged grossly. Lastly, the soft tissue sarcoma from Patient 5 showed moderate microscopic tumor necrosis (<50%) in response to treatment but also remained unchanged in size. Patients 2 and 6 both showed no tumor necrosis grossly post treatment and no tumor necrosis or cell death on histopathologic evaluation following resection. Patient 7 achieved a partial remission over time despite having no microscopic evidence of necrosis in the post-treatment biopsy sample.

**Table 4:** Patient response and post-treatment histopathology

Patient	Response	Histopath pre-treatment	Histopath post treatment	Post-treatment biopsy
1	CR	10% tumor necrosis, no individual cell death	20% necrosis, coagulation and edema	Needle core
2	SD	no necrosis or individual cell death	no evidence of tumor cell death	Excisional
3	SD	<5% necrosis with rare individual cell death	60% necrosis with hemorrhage	Excisional
4	SD	no necrosis or individual cell death	40–50% necrosis and hemorrhage with moderate to severe skin necrosis	Excisional
5	SD	no necrosis and moderate cell death	Less than 50% multifocal mild hemorrhage and necrosis	Excisional
6	SD	no necrosis or individual cell death	no necrosis or individual cell death	Excisional
7	PR	no necrosis or individual cell death	no necrosis or individual cell death	Excisional

### Adverse events

All study related adverse events were considered mild (Table 5 - grade 1, *n* = 10; grade 2, *n* = 5) and generally did not require intervention with supportive care or concomitant medications. Skin/tumor ulceration over the treated area was the primary clinical effect observed in all dogs.

**Table 5:** Patient cutaneous and systemic adverse events

Patient	Cutaneous adverse events	Systemic adverse events
1	Grade 1 erythema; grade 2 skin ulceration	None
2	Grade 2 skin ulceration	Grade 1 anemia
3	Grade 1 skin ulceration	None
4	Grade 2 skin ulceration	Grade 2 ALT elevation; Grade 1 CK elevation
5	Grade 2 skin ulceration	Grade 1 anemia
6	Grade 1 edema and swelling	None
7	Grade 1 erythema and grade 1 tumor necrosis	Grade 1 nausea; Grade 1 lethargy

#### Skin adverse events

Nine adverse events confined to the tumor or surrounding tissues occurred. Erythema, skin ulceration, tumor necrosis and swelling/edema were observed all occurred following NIR laser application.

#### Systemic adverse events

Six systemic adverse events were noted. In all cases, causality with either nanorod administration or laser application could neither be definitively confirmed nor completely ruled out. These included anemia, ALT elevation, CK elevation, nausea and lethargy. Patient 4 experienced a grade 1 CK elevation possibly attributable to tissue damage following NIR laser applications and a grade 2 ALT elevation of unknown etiology. Patient 7 experienced grade 1 nausea and lethargy the day following NIR laser application, which could have been secondary to laser application and subsequent tissue damage or the sedation from the procedure. This patient recovered from both of the adverse events within a 24 h time period. Finally, two patients developed a grade 1 anemia while on study that could have been related to study procedures, although anemia of chronic disease and pre-existing conditions such as hypothyroidism could not be ruled out as the primary causes.

## Discussion

Photodynamic therapy (PDT) has been used in human medicine for decades primarily to treat superficial skin lesions. Major hurdles for the continued success of this treatment modality are specificity of targeting tumors while avoiding toxicity to normal tissues and adverse events related to photosensitizers. Photothermal therapy (PTT) is a therapeutic modality used in human cancer in which intratumoral heating is used to kill tumors through application of a light source specific for the thermal agent. In general, such agents are on the nanoscale given the enhanced permeability and retention effect observed with particles in a certain size range (typically 20–300 nm) [8, 17]. Molecules in this range preferentially accumulate in tumor tissue that typically possesses a disorganized, leaky vasculature and poor lymphatic drainage, different properties as compared to regular blood vessels, such as poor lymphatic drainage and a disorganized, leaky vasculature, promoting this selectivity. The agents used for PTT include gold nanorods (NRs), gold nanoshells, graphene and graphene oxide. Gold NRs have been identified as an optimal nanoparticle as they may be synthesized in bulk, have broadly tunable plasmon resonance and exhibit absorption coefficients 10^4^–10^6^-fold higher than conventional organic fluorochromes typically used for photodynamic therapy [2, 3]. The Surface-Enhanced, Resonant Raman Scattering (SERS) effect created by coupling gold NRs with visible and NIR-absorbing molecules results in enhanced capacity for PTT induced tumor ablation [3].

Multiple methods currently are under investigation to exploit the unique property of SERS NRs to passively collect into tumors [18]. Early in vitro work demonstrated that SERS NRs can be linked with specific monoclonal antibodies to target antigens overexpressed in cancer cells. When exposed to both malignant and non-malignant epithelial cell lines, the SERS NRs showed preferential binding to malignant cell lines that subsequently caused cell death post irradiation at the appropriate spectrum [8]. Recent in vivo work in mouse models with xenograft tumors, which receive IV NRs and 5 min of heating to 70 degrees Celsius with an 810 nm laser, showed destruction of all tumors and durable response for up to 50 days [13]. In addition, gold NRs can be coated with a thermo-sensitive shell that includes dispersed drug molecules that when heated release drug in a targeted area [19]. The synergistic effects of heat and chemotherapy can be quite significant and thus warrant further investigation to allow for maximum efficacy of the PTT effect while potentially reducing toxicity associated with systemic chemotherapy administration [20].

Based on the published data suggesting both safety and efficacy of SERS NRS in the treatment of tumors in laboratory animals, the purpose of this clinical trial was to confirm these findings in a large animal model of cancer that more closely recapitulates the natural evolution of tumors. Therefore, we used dogs with spontaneous tumors in a pilot study setting to evaluate the feasibility and safety of SERS NRs administration combined with NIR diode laser application. A secondary objective of the study was to determine if this therapeutic approach had potential for clinical benefit as assessed by response to therapy. We predicted that the treatment would be safe and associated with a limited adverse event profile, and that most dogs would derive clinical benefit from participation in the study.

Administration of NRs and the subsequent NIR treatment laser treatment were well tolerated by all patients enrolled in this study. Although the number of dogs treated was small, a diverse set of breeds was represented and the size ranged from 9.1 to 42.1 kg. The reported adverse events were expected and related to the extreme intratumoral heating resulting in local cutaneous reactions, swelling, edema and redness. These were self-limiting and nearly all had resolved within 14 days. One of the challenges encountered was the difficulty in achieving consistent and even heating within the treated tumors. In most cases, the tumors developed specific hot spots within 10–20 s of NIR application and effectively moving the laser to evenly distribute the heating was affected by tumor size, tumor shape, and to some degree, operator proficiency. It is likely that this would improve over time with continued experience using the NIR. Additionally, the ability to adjust the circumference of NIR exposure would reduce the need to continually move the laser and thus reduce the development of intratumoral hot spots.

In this study, clearance and processing of the SERS NRs appeared relatively uniform with the exception of a single outlier. Dogs given a dose of 10 mg/kg NRs showed both serum and plasma clearance at 72 h to under 12 μg/ml except in one patient who had a 50% reduction of plasma and serum levels (192 μg/ml immediately after administration to 97μg/ml 72 h later). It is uncertain at this point what might have contributed to this occurrence but changes in either absorption or clearance due to neoplasia, concurrent comorbidities or idiosyncratic causes are all potential etiologies. Importantly, all dogs treated with the NIR laser at 72 h that had detectable NR plasma levels exhibited no additional adverse events when compared to those dogs with undetectable levels. However, as this was a pilot study, the safety threshold for highest potential NR plasma concentration during NIR laser treatment was not determined.

There was no correlation between residual circulating plasma NR levels and response to treatment (Tables 2, 4) supporting the notion that intratumoral accumulation and trapping of the NRs occurred by 72 h post dosing, permitting the observed NIR induced heating to occur.

Two dogs enrolled in this study experienced an objective response to therapy (1 PR, 1 CR). The remaining dogs had stable disease over the 14 day period of study evaluation. When the histopathologic results of dogs experiencing SD versus PR/CR were compared, there appeared to be no differences in the degree of treatment induced necrosis, coagulation and edema. For example, biopsies from dogs 3 and 5 that experienced SD demonstrated 60% and 50% necrosis and hemorrhage, respectively. In contrast, biopsies from dogs 1 (CR) and 7 (PR) had 20% and 0% necrosis and hemorrhage. The authors recognize that the relative amount of necrosis and hemorrhage in the pre treatment samples may not be an accurate representation of the entire tumor, given inherent intra-tumoral heterogeneity. As 6/7 tumors were removed in their entirety, intra tumor heterogeneity in the post treatment samples was minimized as much as possible.

Although all tumors were treated to at least 40 °C, there was a wide range of temperatures achieved during NIR application (from 40 to 110 °C). Time to minimum temperature of 40 °C varied greatly among patients and the authors hypothesize this is likely due to a variety of factors including vascularity, accumulation of NRs in tumors and potentially tumor type. Interestingly, higher temperatures did not appear to correlate with an enhanced induction of necrosis or hemorrhage. Further studies are necessary to determine factors that might be associated with response to therapy, such as in combination with chemotherapy, in order to maximize tumor necrosis.

The limitations of this study are similar to most phase 1 trials including a small non-uniform sample size of patients of varied tumor type and prior therapeutic interventions. Additionally, dose escalation of the NRs was not performed to interrogate a possible link between dose and response to therapy. Dogs were only treated once and it is possible that additional treatments may have enhanced response by promoting further intratumoral accumulation of NRs and thus enhancing the photothermal effect. Lastly, most tumors (5/7) were removed in their entirety at the conclusion of the brief study and another tumor was removed a short time after the study, making it difficult to draw meaningful conclusions regarding clinical benefit from the PTTT. It would have been ideal to allow tumors to be removed after PD was observed; however, the study was designed as a pilot trial with safety as one of the primary endpoints and the intention was not to delay standard of care therapy to patients that were enrolled.

## Conclusions

SERs coated gold NRs are safe for parenteral administration to dogs and the application of NIR treatment following NR administration may hold potential for clinical benefit in dogs with solid tumors. Expansion to a larger cohort of dogs affected with a variety of neoplastic diseases than those enrolled in this pilot study is warranted to truly define the therapeutic potential of this approach. Opportunities exist to combine NR/NIR therapy with other standard treatment modalities such as chemotherapy and immunotherapy to maximize clinical benefit.

## Abbreviations

AEs: Adverse events
ALP: Alkaline Phosphatase
ALT: alanine aminotransferase
AST: aspartate aminotransferase
BUN: blood urine nitrogen
CB: clinical benefit
CBC: complete blood count
CR: complete response
CT: computerized tomography
FS: female spayed
GI: gastrointestinal
IAUCUC: Institutional Animal Care and Use Committee
IV: intravenous
MCT: mast cell tumor
Ml: milliliter
MN: male neutered
NIR: near-infrared
NR: nanorod
NSAID: non-steroidal anti-inflammatory
ORR: overall response rate
PD: progressive disease
PDT: photodynamic therapy
PEG: polyethyleneglycol
PR: partial response
SD: stable disease
SERS: Surface-Enhanced, Resonant Raman Scattering
STS: soft tissue sarcoma
μg: microgram
ULN: upper limit of normal
UV/Vis: ultraviolet visible
VCOG-CTCAE: veterinary cooperative oncology group common terminology criteria for adverse events
